# Treatment and restoration of adult dentoalveolar trauma: A clinical case report

**DOI:** 10.4317/jced.52990

**Published:** 2016-12-01

**Authors:** Blanca Serra-Pastor, Miguel Penarrocha-Diago, María Penarrocha-Diago, Rubén Agustín-Panadero

**Affiliations:** 1 Postgraduate student in Prosthodontics. Department of Buccofacial Prostheses. University Complutense of Madrid. Spain; 2 Chairman of Oral Surgery, Stomatology Department, Faculty of Medicine and Dentistry, University of Valencia, Spain; 3 Full Professor of Oral Surgery, Stomatology Department, Faculty of Medicine and Dentistry, University of Valencia, Spain; 4 Associate Professor of the Department of Stomatology. Faculty of Medicine and Dentistry, Valencia University, Spain

## Abstract

Adult dentoalveolar trauma most often occurs in the context of sports activities and traffic accidents. Coronal fractures are the most common type of lesion, followed by tooth luxation. We present the case of a 25-year-old woman who suffered alveolar bone damage and coronal fractures of the upper incisors, with extrusive luxation of the right central incisor, as the result of a fall. On the first visit, manual reduction of the buccal plate was carried out under local anesthesia, with repositioning of the right central incisor and splinting to the neighboring teeth. Composites were used to restore the coronal fractures. After one month, both upper central incisors and the right lateral incisor were subjected to endodontic treatment. Internal bleaching of the right lateral incisor was also carried out, due to pigmentation secondary to pulp necrosis. At follow-up 5 months later, the alveolar bone fracture was seen to have healed. Definitive anterior restorative treatment with porcelain veneers was therefore carried out. After two years the patient remains asymptomatic and in good dental condition.

** Key words:**Dental trauma, extrusive luxation, dento-alveolar fracture, esthetic restoration.

## Introduction

Adult dentoalveolar trauma is a common result of falls, sports activities, traffic accidents, etc. Although the injuries vary depending on the type, location and direction of the impact, coronal fractures are the most frequent presentation, representing 65-75% of all such dental traumatisms, followed by tooth luxation (8-20%) (1,2).

The management of such traumatisms should follow a series of protocols established for each type of situation. The prognosis in turn is largely dependent upon the time elapsed from injury (3-10).

We describe the treatment and prognosis of an adult patient with alveolar bone fracture and multiple tooth fractures, together with extrusive luxation of the upper right central incisor. The patient signed the correspondent informed consent for the publication of this clinical case report.

## Case Report

**Figure 1:**
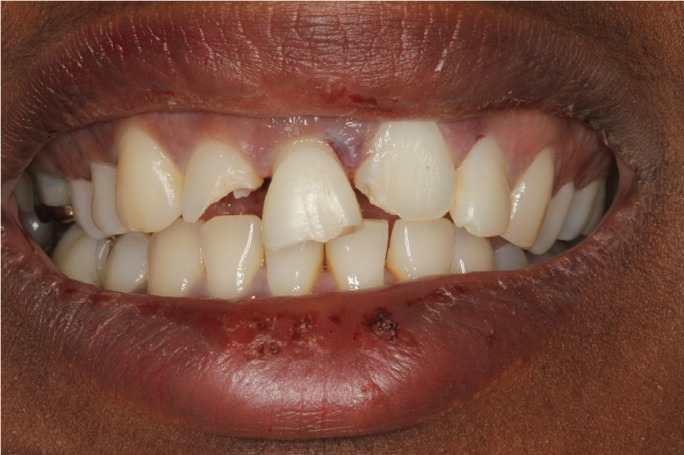
Initial intraoral view showing the coronal fractures of the incisors and extrusive luxation of the upper right central incisor.

**Figure 2:**
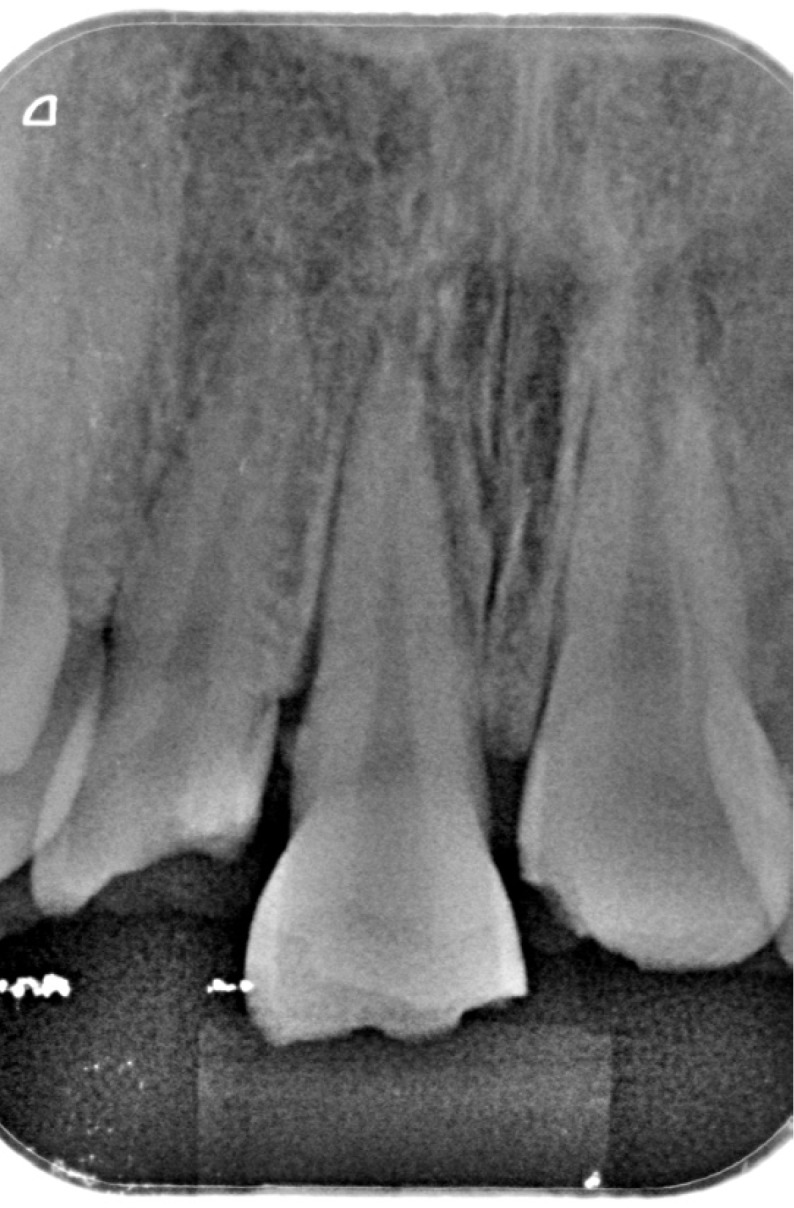
Periapical X-ray view of the upper central incisors, showing widening of the periodontal ligament of the upper right central incisor secondary to extrusive luxation.

**Figure 3:**
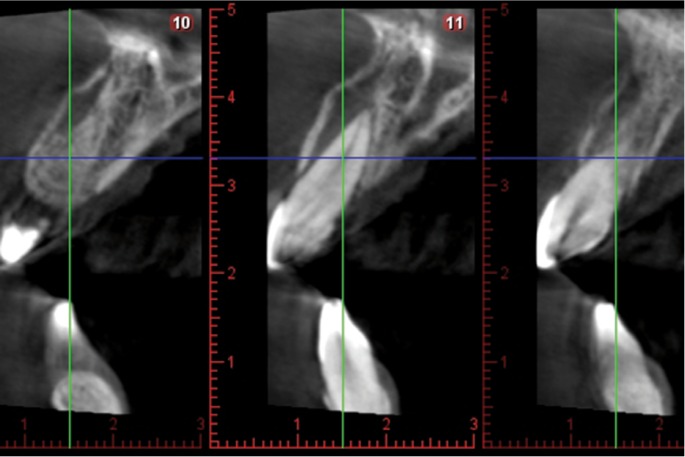
CBCT view showing of the upper central incisors, showing the alveolar fracture of the upper anterior buccal plate.

A healthy 25-year-old woman visited our dental clinic 24 hours after an accidental fall. The physical examination revealed contusion of the lower lip, uncomplicated coronal fracture of all four upper incisors (Fig. 1), and extrusive luxation of the upper right central incisor, which was also confirmed by the X-ray study (Fig. 2). Alveolar fracture of the upper anterior buccal plate was identified by cone beam computed tomography (CBCT) (Fig. 3) (1,3,4).

On the first visit and under local anesthesia, we repositioned the upper right central incisor within its socket, followed by splinting to the neighboring teeth with wire and composite material (5,6). Provisional reconstruction of the coronal fractures with composite was also carried out on the same visit. Antibiotic treatment was prescribed in the form of amoxicillin 875 mg and clavulanic acid 125 mg (Augmentine®, GSK,80 G, Brentford, Middlesex, United Kingdom), together with analgesia in the form of ibuprofen 600 mg (Normon, Madrid, Spain), during one week (1,3,5).

**Figure 4:**
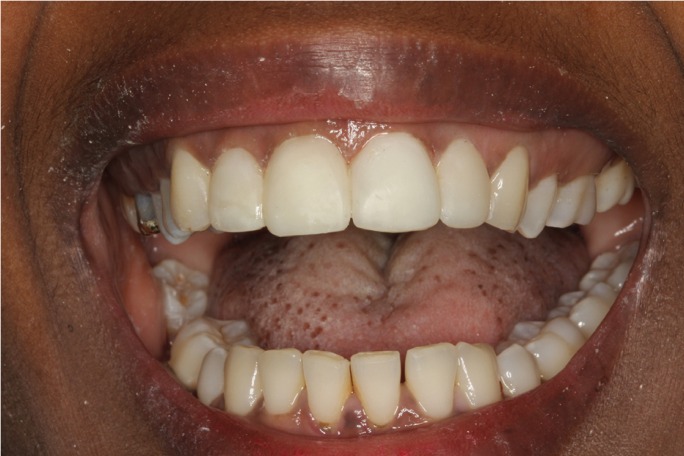
Provisional composite veneers of the four upper incisors.

Three weeks later vitality testing using a tetrafluoroethane spray (Pharmaethyl®, Septodont, Saint-Maur-des-Fossés, France) yielded negative results with both upper central incisors and the right lateral incisor. Endodontic treatment of these teeth was sub-sequently carried out, with internal bleaching of the upper right lateral incisor using 35% hydrogen peroxide (Opalescence Endo; Ultradent, South Jordan, UT, USA), due to discoloration secondary to pulp necrosis. Internal bleaching lasted four weeks, after which composite veneers were prepared (Filtek Supreme XTE; 3M ESPE, St. Paul, MN, USA) for all four upper incisors in order to improve the esthetic outcome (Fig. 4).

Following stabilization of the clinical and esthetic condition of the patient, and after a 5-month waiting period, CBCT evaluation of the alveolar bone fractures confirmed healing of the buccal plate.

**Figure 5:**
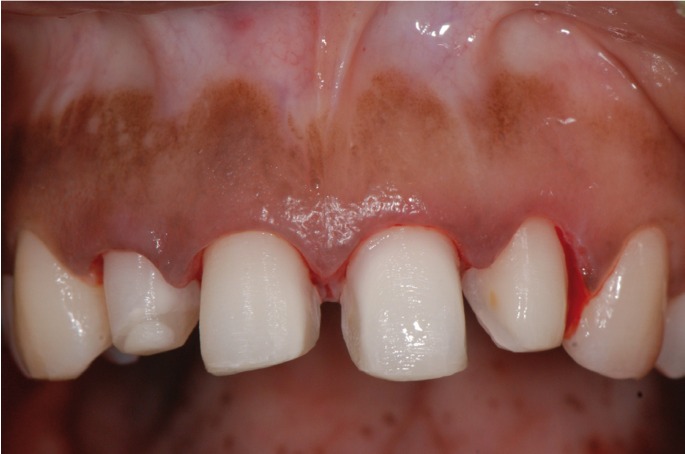
Preparation for veneerson the four upper incisors.

**Figure 6:**
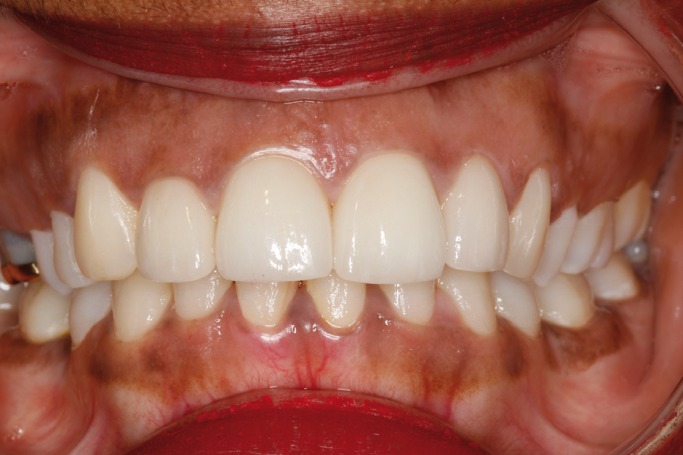
Intraoral view of the porcelain veneers on the upper incisors.

**Figure 7:**
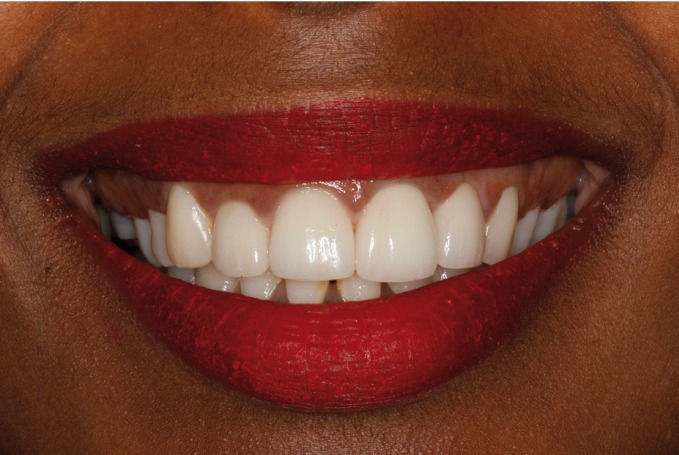
Extraoral view of the porcelain veneers on the upper incisors.

As final restoration, esthetic tests were made for the fitting of lithium disilicate veneers (IPS e.max Press; Ivoclar Vivadent, Schaan, Liechtenstein) on the four upper incisors. Preparation for veneers with a buccal thickness of 0.5 mm and a curved chamfer of 0.3-0.5 mm was carried out (Fig. 5), followed by imprinting with triple-zero retraction thread (Ultrapak; Ultradent, South Jordan, UT, USA) and polyether (Impregum; 3M ESPE, St. Paul, MN, USA) as imprint material. The veneers were cemented using an adhesive technique (Variolink Veneer; Ivoclar Vivadent, Schaan, Liechtenstein), and resulted in a more natural smile (Figs. 6,7).

After two years the patient remains asymptomatic, with no signs of root resorption or ankylosis of the damaged teeth.

## Discussion

Regarding the epidemiology of dental trauma in adults, Robertson et al. found the most frequent lesions to be coronal fractures (65-75% of all cases), followed by tooth luxation (8-20%) (2). The patient presented coronal fractures and the luxation of one of the teeth, with fractures of the upper alveolar processes.

In relation to the treatment of extrusive luxation, Oikarinen *et al.* (6) concluded that splinting with flexible wire and composite affords adequate lateral support for the fixation of luxated teeth, and allows some vertical flexibility – thereby contributing to improve periodontal healing of the affected teeth.

In our case, three of the damaged teeth were subjected to endodontic treatment because of pulp necrosis. According to the literature, 28% of all coronal fractures with luxation end in pulp necrosis (7-9). Robertson and Andreasen concluded that periodontal ligament damage caused by luxation significantly increases the risk of necrosis (8). However, Wang *et al.* (9) reported that intrusive luxation is associated to a higher incidence of pulp necrosis than extrusive luxation, since pulp circulation is more affected in the former case.

Lastly, with regard to the long-term outcome, the literature suggests that root resorption in extrusive luxation is rare, occurring in 5% of those teeth that are repositioned shortly after trauma (10). In our case, after two years of follow-up, the patient remains asymptomatic, with no signs of root resorption or ankylosis of the damaged teeth.

In conclusion, both treatment and restoration in our patient proved successful, with resolution of the dentoalveolar fractures and recovery of good esthetics in the anterior sector.
